# Hippocampal sparing radiotherapy for glioblastoma patients: a planning study using volumetric modulated arc therapy

**DOI:** 10.1186/s13014-016-0695-6

**Published:** 2016-09-08

**Authors:** Jan Hofmaier, Steffi Kantz, Matthias Söhn, Oliver S. Dohm, Stefan Bächle, Markus Alber, Katia Parodi, Claus Belka, Maximilian Niyazi

**Affiliations:** 1 Klinikum der Universität München, Klinik und Poliklinik für Strahlentherapie und Radioonkologie, Marchioninistraße 15, D-81377 München, Germany; 2 Eberhard Karls Universität Tübingen, Universitätsklinik für Radioonkologie, Tübingen, Germany; 3 Department of Oncology, Aarhus Universitet, Aarhus C, Denmark; 4 Fakultät für Physik der LMU München, Lehrstuhl für Experimentalphysik - Medizinische Physik, Munich, Germany

**Keywords:** Glioblastoma, Hippocampal sparing, VMAT

## Abstract

**Background:**

The purpose of this study is to investigate the potential to reduce exposure of the contralateral hippocampus in radiotherapy for glioblastoma using volumetric modulated arc therapy (VMAT).

**Methods:**

Datasets of 27 patients who had received 3D conformal radiotherapy (3D-CRT) for glioblastoma with a prescribed dose of 60Gy in fractions of 2Gy were included in this planning study. VMAT plans were optimized with the aim to reduce the dose to the contralateral hippocampus as much as possible without compromising other parameters. Hippocampal dose and treatment parameters were compared to the 3D-CRT plans using the Wilcoxon signed-rank test. The influence of tumour location and PTV size on the hippocampal dose was investigated with the Mann–Whitney-*U*-test and Spearman’s rank correlation coefficient.

**Results:**

The median reduction of the contralateral hippocampus generalized equivalent uniform dose (gEUD) with VMAT was 36 % compared to the original 3D-CRT plans (*p* < 0.05). Other dose parameters were maintained or improved. The median V30Gy brain could be reduced by 17.9 % (*p* < 0.05). For VMAT, a parietal and a non-temporal tumour localisation as well as a larger PTV size were predictors for a higher hippocampal dose (*p* < 0.05).

**Conclusions:**

Using VMAT, a substantial reduction of the radiotherapy dose to the contralateral hippocampus for patients with glioblastoma is feasible without compromising other treatment parameters. For larger PTV sizes, less sparing can be achieved. Whether this approach is able to preserve the neurocognitive status without compromising the oncological outcome needs to be investigated in the setting of prospective clinical trials.

## Background

Glioblastoma multiforme (GBM) constitutes one of the most frequent and most lethal primary brain tumours, with an annual incidence rate of approximately 3 in 100,000 people in the US and only about 36 % of patients surviving the first year after initial diagnosis [1]. The standard treatment approach for GBM is trimodal, with maximal safe resection, if feasible, followed by radiochemotherapy and temozolomide-based maintenance chemotherapy. However, radiotherapy of the brain may have side-effects, which include radiation necrosis, cognitive impairment, and others [2–5]. The dose to the hippocampus is associated with radiation induced memory impairment [6–9]. Since modern radiotherapy techniques such as intensity-modulated radiotherapy (IMRT) and volumetric modulated arc therapy (VMAT) allow for the delivery of highly conformal dose distributions, the idea arose to selectively spare the hippocampus during brain radiotherapy [10]. In this planning study, the potential to spare the contralateral hippocampus in radiotherapy for glioblastoma using the VMAT technique is investigated for 27 patients. Resulting dose volume histogram (DVH) statistics are compared to clinical standard 3D-conformal radiotherapy (3D-CRT) plans.

## Methods

Datasets of 27 patients who had received 3D-CRT for glioblastoma at the department of radiation oncology at the Ludwig-Maximilians University of Munich (LMU) between 2010 and 2012 were included in this planning study. For all patients, the prescription to the planning target volume (PTV) was 60Gy in 30 fractions. The patient characteristics are shown in Table 1. Patients were positioned in supine position and immobilized with a thermoplastic mask. Normal structures like the eyes, the lenses, the optic nerves, the chiasm, the brain stem were contoured and designated as organs-at-risk (OARs). Expanded contours were created with safety margins of 3 mm around the brain stem and the chiasm and 5 mm around the optic nerve. The hippocampus was contoured on the T1 MRI sequence as described by Chera et al. [11]. The VMAT plans were optimised in the research treatment planning system (TPS) Hyperion V2.44 (equivalent to Elekta Monaco 5.1) which relies on the XVMC algorithm (X-ray voxel Monte Carlo [12]) for dose calculation. All VMAT plans were generated for a 6MV Elekta Axesse linear accelerator (LINAC) equipped with an Agility multileaf-collimator (Elekta, Stockholm, Sweden). The original clinical 3D-CRT plans were generated in the TPS Oncentra Masterplan (Elekta, Stockholm, Sweden) using a pencil beam dose algorithm. 3D-CRT plans were created for Siemens LINACs (Oncor (19 patients), Primus (4) and Mevatron M (4)) equipped with an 80 or 58 leaves MLC. Since the aim of this study was a comparison of clinical planning strategies rather than a technical comparison between VMAT and 3D-CRT, the use of two different TPS and dose calculation algorithms is not considered to bias the results significantly. In both systems, planning was performed independently. We supposed that slight differences between the beam models only have small influence on the optimal plan a dosimetrist can create using these models. The dose grid was 3×3×3mm^3^ for both VMAT and 3D-CRT plans.

**Table 1:** Patient characteristics

Number of patients	27
Male	17 (63.0 %)
Female	10 (37.0 %)
Age	
*Median*	65 years
*Range*	28–82 years
Tumour localisation	
*Left*	15 (55.6 %)
*Right*	8 (29.6 %)
*Bilateral*	4 (14.8 %)
Tumour location	
*Frontal*	4
*Temporal*	12
*Parietal*	3
*Occipita*l	1
*Fronto-temporal*	4
*Temporo-parietal*	1
*Fronto-parietal*	1
*Multifocal*	1
PTV volume	
*Median*	393.8ccm
*Range*	169.1–608.0ccm
Contralateral hippocampus volume	
*Median*	2.3ccm
*Range*	1.4–3.1ccm

### VMAT planning strategy

**Fig. 1:**
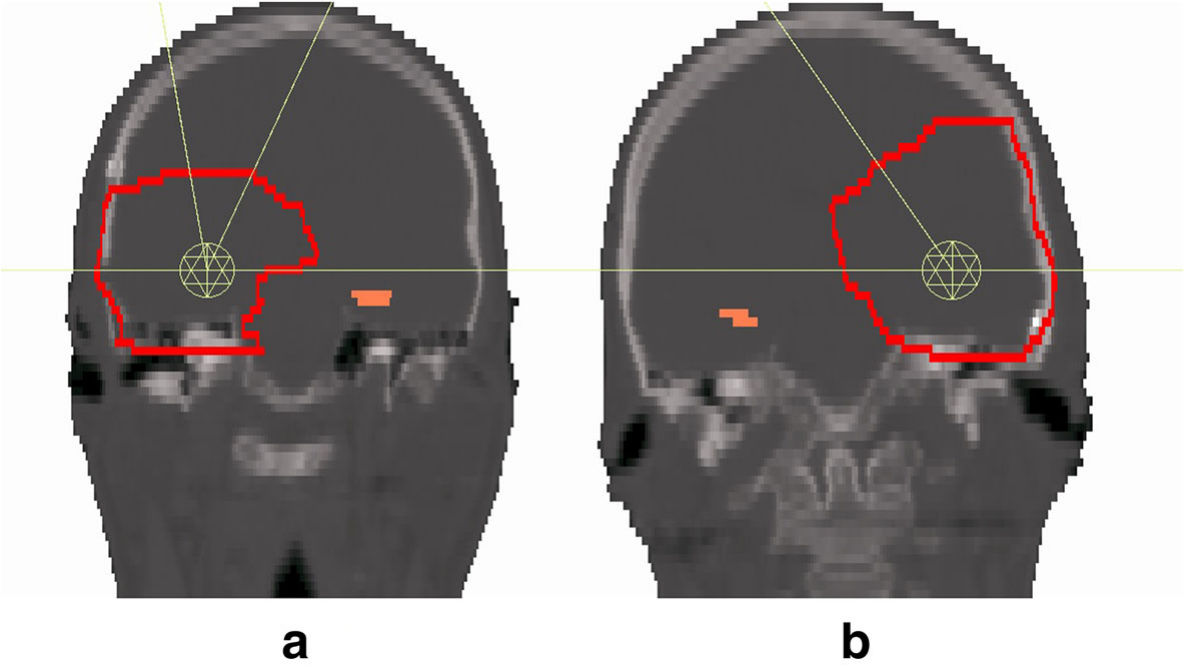
Coronal view of the arc setup*.* **a** Two full coplanar arcs (*horizontal line*) and one arc from gantry angle 0 to 180° at couch angle 55° were sufficient in most cases (16 patients). **b** For the remaining 11 patients, an additional non-coplanar arc was introduced

The VMAT plans were realized with two full 360° coplanar arcs in clockwise and counter clockwise directions and one non-coplanar arc with a length of 180° at couch angle 55° or −55°, depending on the location: The couch angle was chosen to minimize the amount of healthy tissue traversed by the beams. The collimator angle for the coplanar arcs was chosen individually according to the angle of the hippocampus in the sagittal plane, which was typically in the range of 50° to 70°. For 11 patients, this approach did not result in clinically acceptable dose distributions, and an additional non-coplanar arc was introduced at an individually chosen angle. The arc setup is illustrated in Fig. 1.

### Optimisation constraints

The optimisation constraints for OARs are summarized in Table 2. The dose to the cochlea was limited to 54Gy when it was not inside or in the direct vicinity of the PTV (11 patients). For the remaining 16 patients, the dose was limited to 60Gy to avoid the appearance of hot spots in the cochlea. The constraint limiting the mean dose to the contralateral hippocampus was adjusted individually for each patient. It was attempted to reduce the dose as much as possible without compromising on the target coverage and the dose limits for other OARs. No attempt was made to spare the ipsilateral hippocampus. For patients with bilateral disease (4 cases), the side bearing a lower tumour volume was the side where the hippocampal dose was reduced. All plans were reviewed by a consultant radiation oncologist and approved as clinically acceptable.

**Table 2:** Optimisation constraints

OAR	Dose constraint
Lenses	Maximum dose ≤ 5Gy
Eyes	Mean dose ≤ 30Gy
Optic nerves	Maximum dose ≤ 54Gy (5 mm expansion: ≤ 56Gy)
Chiasm	Maximum dose ≤ 54Gy (3 mm expansion: ≤ 56Gy)
Brainstem	Maximum dose ≤ 54Gy (3 mm expansion: ≤ 56Gy)
Cochlea	Maximum dose ≤ 54Gy/60Gy
Hippocampus	Chosen individually for each patient

### Treatment plan evaluation

To assess the plan quality, the following metrics were calculated both for the original 3D-CRT and the new VMAT plans. The percentage of a structure receiving at least the dose x is calculated with the formula

$$ Vx=\frac{V_{T,x}}{V_T} $$

where *V*_*T*,*x*_ is the volume of the structure receiving at least the dose x and *V*_*T*_ is the volume of the structure, both in absolute volume units. The index was used to evaluate the coverage of the PTV with the prescribed dose as well as for the evaluation of the exposure of the brain and the hippocampus. The homogeneity index HI as proposed in the ICRU report 83 [13] is calculated with the formula

$$ HI=\frac{D_{2\%}-{D}_{98\%}}{D_{50\%}} $$

where *D*_*x* %_ is the dose received by x% of the target volume. For perfect homogeneity *D*_2 %_ and *D*_98 %_ are equal and HI takes the value 0.0. For inhomogeneous dose distributions, it takes values >0.0. The conformity index with respect to the isodose x, *CI*_*x*_, was calculated as proposed by Paddick [14]:

$$ C{I}_x=\frac{V_{T,\ x}}{V_T}\cdotp \frac{V_{T,x}}{V_x} $$

Where *V*_*T*_ and *V*_*T*,*x*_ are calculated for the target volume and *V*_*x*_ denotes the volume receiving the dose x, including also volumes outside the target volume (in absolute volume units). For perfect conformity, *CI*_*x*_ takes the value 1.0. Less conformal dose distributions result in values <1.0. In this study, *CI*_*x*_ was evaluated with respect to the isodoses 100 % and the 95 % of the prescribed dose. To quantify the dose to the hippocampus, the generalized equivalent uniform dose (gEUD) was used. This concept was introduced by Niemierko [15]. In this approach, two heterogeneous dose distributions are assumed to be equivalent when they have the same biological effect. For normal tissues the gEUD is calculated:

$$ gEUD={\left({\displaystyle \sum_j}{\nu}_j{D}_j^k\right)}^{\raisebox{1ex}{$1$}\!\left/ \!\raisebox{-1ex}{$k$}\right.} $$

where the empirical parameter k corresponds to the strength of the volume effect (for *k* → ∞ the gEUD is the maximum dose, for k = 1 it corresponds to the mean dose). For the hippocampus, k = 12 was used as previously described [16].

Doses are reported in absolute planning dose, if not stated otherwise. For the hippocampus also EQD2 (equivalent dose in 2Gy fractions) values have been calculated, since these values are often used in the literature.

### Statistical analysis

For the statistical comparison of the 3D-CRT and the VMAT plans implementations of statistical tests in the software Mathematica, Version 9.0 (Wolfram Research, Inc., Champaign, IL) were used. When paired samples were to be compared, as for the comparison of treatment parameters such as target indices and the dose to the contralateral hippocampus between 3D-CRT and VMAT the Wilcoxon signed-rank test was used, since a Kolmogorov-Smirnov test revealed that parametric conditions were not fulfilled in all cases. When different samples were compared, which was the case when the influence of the tumour localisation on the hippocampal dose was investigated, the Mann–Whitney *U* test was used. To assess the correlation between the PTV size and the hippocampal dose, Spearman’s rank correlation coefficient ρ and corresponding *p*-values were calculated. A *p*-value of 0.05 was considered significant.

## Results

**Fig. 2:**
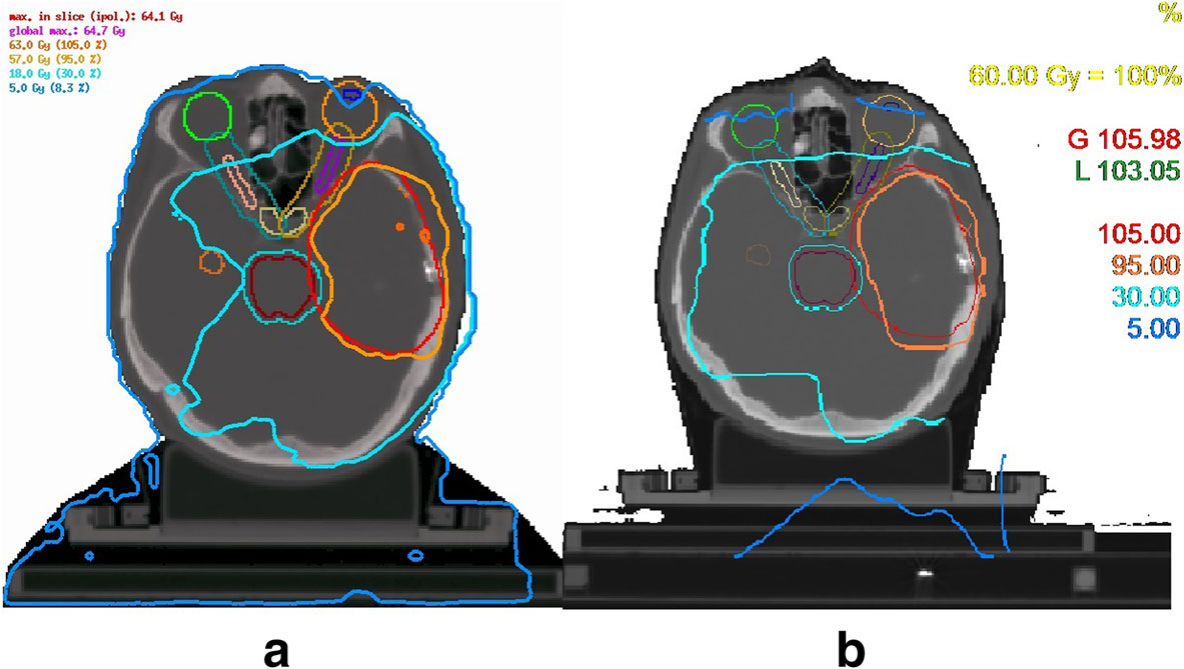
Comparison of VMAT (**a**) and 3D-CRT (**b**) dose distributions for one patient*.* OARs are shown with their avoidance margins. Dose constraints for optic nerve (*violet*), chiasm (light brown, only the 3 mm expansion is visible in this slice) and the brain stem (*green*) prevent the 95 % isodose from completely covering the PTV (*red*). The reduced dose to the contralateral hippocampus (*yellow*) can be seen in the 30 % isodose line. The 5Gy isodose line shows that the dose to the lenses was limited to 5Gy

In Fig. 2, a comparison of the isodose lines in the same transversal slice between the VMAT and the 3D-CRT plans is shown for one exemplary patient case. The reduction of the hippocampal dose exposure can be seen in the 30 % (=18Gy) isodose line.

### Target indices

In Table 3 the target indices for the 3D-CRT and the VMAT plans are shown. The VMAT plans provided a better homogeneity index and a better conformity index with respect to the 95 % isodose. The median HI with VMAT was 0.15, while for 3D-CRT it was 0.18. The median *CI*_95 %_ was 0.80 for VMAT compared to 0.70 for 3D-CRT. Both differences were statistically significant (*p* < 0.01). No significant differences between the two techniques were observed according to target coverage and conformity index with respect to the 100 % isodose.

**Table 3:** PTV indices, dose to the brain and to the contralateral hippocampus

	3D-CRT/median (range)	VMAT/median (range)	*p*-value
PTV indices			
*V*_95 %_	0.88 (0.79–0.97)	0.91 (0.79–0.99)	NS (*p* = 0.05)
*V*_100 %_	0.53 (0.17–0.75)	0.56 (0.38–0.84)	NS (*p* = 0.18)
HI	0.18 (0.09–0.24)	0.15 (0.06–0.21)	*p* < 0.01
*CI*_95 %_	0.70 (0.32–0.78)	0.80 (0.75–0.91)	*p* < 0.01
*CI*_100 %_	0.48 (0.15–0.70)	0.55 (0.36–0.78)	NS (*p* = 0.09)
Brain			
*Mean dose (Gy)*	35.7 (23.7–45.0)	34.0 (24.2–40.3)	*p* < 0.01
*V*_12*Gy*_ *(%)*	89.6 (53.5–98.5)	89.2 (70.7–97.3)	*p* < 0.01
*V*_30*Gy*_ *(%)*	58.7 (31.3–81.0)	48.2 (27.2–62.4)	*p* < 0.01
*V*_45*Gy*_ *(%)*	40.0 (19.0–56.3)	32.9 (17.7–47.7)	*p* < 0.01
Contralateral hippocampus			
*Mean dose (Gy)*	33.1 (12.1–50.1)	14.7 (8.1–40.3)	*p* < 0.01
*Mean EQD2 (Gy)*	25.7 (7.3–46.0)	9.2 (4.6–33.7)	*p* < 0.01
*Max dose (Gy)*	39.6 (30.1–59.9)	26.7 (12.6–62.3)	*p* < 0.01
*Max EQD2 (Gy)*	32.9 (22.6–59.9)	19.3 (7.6–63.5)	*p* < 0.01
*gEUD (Gy)*	36.3 (24.3–56.1)	23.1 (10.8–56.0)	*p* < 0.01
*V*_10*Gy*/5.8*Gy*(*EQD*2)_ *(%)*	100. (44.3–100.)	100. (14.2–100.)	*p* < 0.01
*V*_15*Gy*/9.4*Gy*(*EQD*2)_ *(%)*	100. (14.4–100.)	32.3 (0.0–100.)	*p* < 0.01
*V*_20*Gy*/13.3*Gy*(*EQD*2)_ *(%)*	100. (8.3–100.)	12.6 (0.0–83.0)	*p* < 0.01
*V*_30*Gy*/22.5*Gy*(*EQD*2)_ *(%)*	70.5 (2.2–100.)	3.4 (0.0–69.0)	*p* < 0.01
*V*_40*Gy*/33.3*Gy*(*EQD*2)_ *(%)*	1.4 (0.0–100.)	0.4 (0.0–61.3)	*p* < 0.01

### Dose to the brain

The difference between the mean brain dose for the two techniques was small but significant (median 34.0Gy for VMAT vs. 35.7Gy for 3D-CRT, *p* < 0.01). Additionally, the percentage of the brain volume receiving 12, 30 and 45Gy was significantly lower for the VMAT technique (*p* < 0.01), the largest differences were observed for the *V*_30*Gy*_ (brain) (median 48.2 % for VMAT vs. 58.7 % for 3D-CRT). Details are shown in Table 3.

### Dose to the contralateral hippocampus

The dose to the contralateral hippocampus is shown in Table 3 PTV indices, dose to the brain and to the contralateral hippocampus. All the calculated parameters (mean dose, maximum dose, gEUD and the percentage of the contralateral hippocampus volume receiving 10, 15, 20, 30 and 40Gy) were significantly improved in the VMAT plans (*p* < 0.01). In particular, the median *V*_20*Gy*_ (hippocampus) improved from 100 % for 3D-CRT to 12.6 % for VMAT and the median *V*_15*Gy*_ (hippocampus) from 100 to 32.2 %. The median gEUD was reduced by 36 % with VMAT.

### Correlation between the size of the PTV and the dose to the hippocampus

Spearman correlation coefficients and *p*-values are shown in Table 4. No significant correlation between the size of the PTV and the hippocampal dose was observed for 3D-CRT. For the VMAT plans, a significant correlation (*p* = 0.01) was found for the hippocampal mean dose, gEUD and *V*_15*Gy*_ (ρ = 0.44,0.47 and 0.41, respectively).

**Table 4:** Correlation of the PTV size with the respective dose to the contralateral hippocampus

Parameter	Spearman’s ρ	*p*-value
*3D-CRT*		
*Mean Dose*	0.21	NS (*p* = 0.30)
*gEUD*	0.33	NS (*p* = 0.10)
*V*_15*Gy*/9.4*Gy*(*EQD*2)_	0.11	NS (*p* = 0.59)
*VMAT*		
*Mean dose*	0.44	*p* = 0.02
*gEUD*	0.47	*p* = 0.01
*V*_15*Gy*/9.4*Gy*(*EQD*2)_	0.41	*p* = 0.01

### Influence of the PTV localisation on the hippocampal dose

In Table 5, the results of the analysis of the influence of the tumour localisation on the hippocampal gEUD are shown. For 3D-CRT, no significant influence of the tumour localisation on the hippocampal dose was observed. For VMAT, a parietal localisation appears to go along with a higher hippocampal dose. The median gEUD to the hippocampus of all VMAT plans for patients with parietal involvement was 37.9 Gy, compared to 21.1Gy for the remaining patients without parietal involvement (*p* < 0.01). For the subgroup of patients with no temporal involvement, the dose to the contralateral hippocampus (median gEUD 31.2 Gy) was higher (*p* = 0.04) than for the patients with temporal involvement (median gEUD 19.5Gy).

**Table 5:** Tumour localisation and hippocampal dose

	Median (range)		*p*-value
3D-CRT			
*Temporal*		*Not temporal*	
gEUD (Gy)	34.3 (26.0–48.6)	40.4 (24.3–56.1)	NS (*p* = 0.09)
*Parietal*		*Not parietal*	
gEUD (Gy)	40.6 (24.3–56.1)	36.0 (26.0–48.0)	NS (*p* = 0.40)
*Frontal*		*Not frontal*	
gEUD (Gy)	38.0 (30.8–48.6)	33.7 (24.3–56.1)	NS (*p* = 0.05)
VMAT			
* Temporal*		*Not temporal*	
gEUD (Gy)	19.5 (10.8–43.1)	31.2 (17.9–56.0)	*p* = 0.04
*Parietal*		*Not parietal*	
gEUD (Gy)	37.9 (25.7–56.0)	21.1 (10.8–43.1)	*p* < 0.01
*Frontal*		*Not frontal*	
gEUD (Gy)	22.4 (10.8–48.0)	24.1 (10.8–56.0)	NS (*p* = 0.86)

## Discussion

The purpose of this planning study is to investigate the feasibility of sparing the contralateral hippocampus in radiotherapy for glioblastoma using VMAT. The hippocampal dose and the whole brain *V*_30*Gy*_ could be significantly improved, while maintaining the same dose constraints for other OARs. The other investigated parameters (target coverage, homogeneity index and conformity index) could be kept stable or even improved (dose homogeneity and conformity, whole brain dose). These results are in agreement with the results found in similar studies by Canyilmaz et al., who compared standard IMRT to hippocampal sparing IMRT and VMAT plans for 20 patients [17], and Marsh et al. who compared standard IMRT plans to IMRT plans with sparing of the contralateral neural stem cell compartment, hippocampus and limbic circuit for 5 patients [18]. The planning strategy in these studies differed from the concept used in our planning study. Both studies used a dose prescription of 46 Gy in 23 fractions followed by a sequential boost of 14Gy in 7 fractions (RTOG consensus) and concluded that a substantial reduction of the dose to the contralateral hippocampus is feasible with the used techniques without compromising other treatment parameters. In contrast to those studies, we also attempted to identify influencing factors for the hippocampal dose exposure. Studies suggest that it is safe to reduce the applied margins in IMRT for glioblastoma [19]. A study by Ali et al. evaluated the effect of reduced PTV margins on hippocampal dose [20]. They compared the standard margin of 2 cm (gross tumour volume (GTV) to clinical target volume (CTV)) with a reduced margin of 8 mm and reported a significant reduction of the bilateral hippocampal dose when applying the reduced margin concept. Concordantly, a significant correlation between the size of the PTV and the dose to the contralateral hippocampus was observed for VMAT in our planning study. This correlation was not significant for the original 3D-CRT plans, which might be explained by the fact that in the standard clinical protocol hippocampal sparing was not a treatment goal, even though it was attempted to create a dose distribution as conformal as possible, but the hippocampus was not designated as an OAR for which the dose exposure should be selectively reduced. This is also a limitation of this study. It is not suited to investigate the question to what extent VMAT is better in sparing the hippocampus compared to 3D-CRT from a purely technological perspective, since the planning objectives were not the same. However, it allows us to draw conclusions about how much reduction of the dose exposure can be achieved clinically, when the standard 3D-CRT planning strategy is replaced by a VMAT approach and hippocampal sparing is incorporated as a planning objective. An unexpected result of our study is that a parietal tumour localisation correlated with a higher hippocampal dose for VMAT, while a temporal localisation correlated with a lower hippocampal dose. A possible explanation may be the used planning strategy - for some angles of the non-coplanar arcs, the contralateral hippocampus is in the field when the target volume is located on the parietal lobe. Given that the subgroup of patients with parietal involvement was small (5 patients), which limits the statistical power of the test and that the *p*-value for the comparison between patients with and without temporal involvement was just under the significance threshold (*p* = 0.04), these particular results should be interpreted with caution. While there have been few studies investigating the feasibility of sparing the hippocampus in radiotherapy for glioblastoma, more research has been carried out concerning hippocampal sparing during whole-brain radiotherapy (WBRT) for patients with brain metastases. Several studies have investigated the feasibility of sparing both hippocampi during WBRT [21–24]. Clinical data suggests that the approach is safe and favourable in terms of neurological outcome [25–27]. For hippocampal sparing WBRT, also the influence of patient positioning on the hippocampal dose exposure has been investigated, and there is data suggesting that an inclined head angle might be beneficial [28]. In our study, it was not investigated whether an inclined head angle is able to further improve hippocampal dose in radiotherapy for glioblastoma, as all patients were positioned in the standard way. In the setting of WBRT for patients with brain metastases, sparing the hippocampus is not expected to affect the therapeutic ratio, since brain metastases rarely occur inside or close to the hippocampus [29, 30]. By contrast, sparing the hippocampus in radiotherapy for GBM is more controversial because of potential involvement of the subventricular zone (SVZ) in tumour genesis [31]. Higher doses to the ipsilateral SVZ have been shown to correlate with improved progression-free survival [32], and no SVZ contact of the tumour volume has been shown to be a predictor for long-term survival [33]. For this reason, only the contralateral hippocampus was spared in this planning study. In the cases with bilateral tumour, no involvement of the SVZ was presumed due to the small tumour volume on the spared side. Whether hippocampal sparing is possible without increasing the risk of relapse is a matter of ongoing debate [34, 35]. Possible benefits for patients with regard to neurological toxicity of radiotherapy for glioblastoma still need to be evaluated in future clinical trials. A retrospective analysis by Bodensohn et al. concluded that sparing might make sense for about 50 % of the patients receiving radiotherapy for glioblastoma [36].

## Conclusions

Using VMAT instead of the standard 3D-CRT planning procedure, a significant reduction of the dose to the contralateral hippocampus (median reduction: 36 %) is feasible. Other treatment parameters can be improved as well or at least be maintained. Whether this approach is able to improve the outcome of patients with glioblastoma needs to be investigated in the setting of prospective clinical trials. A larger size of the PTV is a predictor for a higher hippocampal dose in the VMAT plans.

## Abbreviations

3D-CRT: Three-dimensional conformal radiotherapy
CI: Conformity index
CTV: Clinical target volume
DVH: Dose-volume histogram
EQD2: Equivalent dose in 2Gy fractions
GBM: Glioblastoma multiforme
gEUD: Generalized equivalent uniform dose
GTV: Gross tumour volume
HI: Homogeneity index
MLC: Multileaf collimator
OAR: Organ-at-risk
PTV: Planning target volume
RTOG: Radiation therapy oncology group
SVZ: Subventricular zone
TPS: Treatment planning system
VMAT: Volumetric modulated arc therapy
WBRT: Whole-brain radiotherapy
XVMC: X-ray voxel Monte Carlo
